# Reproducible culture and differentiation of mouse embryonic stem cells using an automated microwell platform^☆^

**DOI:** 10.1016/j.bej.2013.05.008

**Published:** 2013-08-15

**Authors:** Waqar Hussain, Nathalie Moens, Farlan S. Veraitch, Diana Hernandez, Chris Mason, Gary J. Lye

**Affiliations:** The Advanced Centre for Biochemical Engineering, Department of Biochemical Engineering, University College London, Torrington Place, London WC1E 7JE, UK

**Keywords:** Stem cell, Bioprocess automation, Stem cell expansion, Neural differentiation, Consistency, Reproducibility

## Abstract

The use of embryonic stem cells (ESCs) and their progeny in high throughput drug discovery and regenerative medicine will require production at scale of well characterized cells at an appropriate level of purity. The adoption of automated bioprocessing techniques offers the possibility to overcome the lack of consistency and high failure rates seen with current manual protocols. To build the case for increased use of automation this work addresses the key question: “can an automated system match the quality of a highly skilled and experienced person working manually?” To answer this we first describe an integrated automation platform designed for the ‘hands-free’ culture and differentiation of ESCs in microwell formats. Next we outline a framework for the systematic investigation and optimization of key bioprocess variables for the rapid establishment of validatable Standard Operating Procedures (SOPs). Finally the experimental comparison between manual and automated bioprocessing is exemplified by expansion of the murine Oct-4-GiP ESC line over eight sequential passages with their subsequent directed differentiation into neural precursors. Our results show that ESCs can be effectively maintained and differentiated in a highly reproducible manner by the automated system described. Statistical analysis of the results for cell growth over single and multiple passages shows up to a 3-fold improvement in the consistency of cell growth kinetics with automated passaging. The quality of the cells produced was evaluated using a panel of biological markers including cell growth rate and viability, nutrient and metabolite profiles, changes in gene expression and immunocytochemistry. Automated processing of the ESCs had no measurable negative effect on either their pluripotency or their ability to differentiate into the three embryonic germ layers. Equally important is that over a 6-month period of culture without antibiotics in the medium, we have not had any cases of culture contamination. This study thus confirms the benefits of adopting automated bioprocess routes to produce cells for therapy and for use in basic discovery research.

## Highlights

• We describe an automated platform for hands-free ESC expansion and differentiation.
• Key bioprocess variables were investigated to optimize culture inductions.
• Cell growth was more consistent with automated ESC expansion than manual culture.
• ESCs expanded on the automated platform maintained high levels of pluripotency.
• Cells expressed βIII-tubulin after successful automated neuronal differentiation.

## 1 Introduction

Embryonic stem cells (ESCs) are currently being evaluated for potential application in a number of diverse areas including regenerative medicine [1], drug discovery and development [2–4] or as routes for delivery of gene therapies [5]. The high level of interest is a consequence of the ability of ESCs to self renew indefinitely and to differentiate into almost every somatic cell type [6–10].

Effective exploitation of ESCs is, however, predicated upon the ability to reproducibly derive, manipulate and efficiently differentiate these cells on a suitable scale at an appropriate level of purity [11–15]. The often reported lack of reproducibility in ESC processing is a consequence of the large number of processing steps involved, generally carried out using largely uncontrolled and manual operations. Previous work in our laboratory and elsewhere has shown that ESCs are particularly sensitive to the microenvironment prevalent during processing [11,16–18]. For example, the effect of physical forces on cells due to fluid flow during pipetting was shown to be highly significant resulting in a large degree of culture variability among similarly trained operators. Automation of ESC handling offers the potential to reduce such variation since applied physical forces may be tightly controlled and applied consistently throughout a culture, and from culture to culture [19].

A number of initial publications on the automation of aspects of ESC processing have appeared in the last few years. The first was that of Joannides and co-workers [20] who modified an existing ‘tissue chopping device’ to create an instrument for the automated mechanical passaging of human ESCs. This work was the first to address the pressing need for standardization of routine, and often variable laboratory work with a view to processing stem cells at scale for therapy. The use of automated liquid handling to address the issue of standardization of physical forces resultant of fluid flow has also been reported [19]. In this case the plating out of both human and murine ESCs using a liquid handling robot was reported along with automated media exchanges. Complete automated passaging of ESCs was precluded by the lack of an integrated centrifuge to facilitate removal of cell dissociating agents. Automated expansion of ESCs in traditional T-flasks has also been reported [21]. It was shown that ESCs can be routinely maintained and expanded in an automated T-flask system yielding cell numbers at the scale required for therapeutic applications. Most recently, murine ESCs have been expanded in microplates on an automated liquid handling platform and further differentiated into cardiomyocytes by embryoid body (EB) differentiation on a separate microfluidic liquid handling workstation [22], which is optimal for high-throughput screening. Manual transfer of cells was required however between the expansion and differentiation stage. Furthermore, none of the platforms described incorporation of the key centrifugation step to facilitate enzymatic passaging of cells. To date then, there remains no report in the literature on the complete, hands-free automation of all key steps in the processing of ESCs including trypsinization, centrifugation and cell differentiation.

**Fig. 1:**
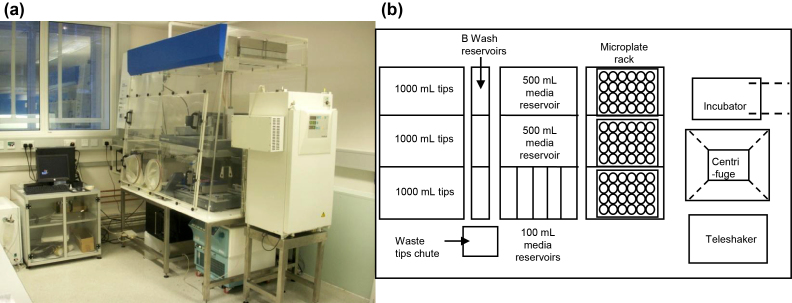
Automation platform for the reproducible culture and differentiation of mouse embryonic stem cells. (a) Photograph of liquid handling robot contained within the Class-2 design biosafety cabinet. (b) Schematic layout of the robotic deck showing access (dashed lines) to the integrated CO_2_ incubator (plate conveyor belt) and microplate centrifuge (via the Tecan RoMa arm).

In this work, we address a key question related to the use of stem cells in drug discovery and future development of the regenerative medicine industry [12]: ‘Can an automated system match the quality of a highly skilled and experienced person working manually?’ Consequently, we describe an integrated automation platform (Fig. 1(a)) for the ‘hands-free’ expansion and differentiation of ESCs. Related to this is a framework for the systematic investigation and optimization of key bioprocess variables in order to define validatable Standard Operating Procedures (SOPs) for automation platform operation. The comparison between automated and manual processing is exemplified by the maintenance of the murine ESC line Oct-4-GiP [23], in 24-well microtitre plates, over eight sequential passages. The subsequent automated and directed neural differentiation of these ESCs is also demonstrated. Our results show that ESCs can be effectively maintained and differentiated in a highly reproducible manner by the automated system described.

## 2 Materials and methods

### 2.1 Routine cell culture and maintenance

The mouse embryonic stem cell line Oct-4-GiP [23] was used throughout this work and was kindly provided by Stem Cell Sciences (Cambridge, UK). The cells were cultured under feeder-free conditions on Iwaki tissue culture treated plastic (SLS, Nottingham, UK) coated with 0.1% (w/v) gelatine (Sigma, Poole, UK). Routine maintenance of the cells was carried out in T-flasks and all other experimental work was carried out in 24-well microplates. For undifferentiated cell growth the culture medium consisted of Glasgow Minimum Essential Medium (GMEM) supplemented with 0.1 mM β-mercaptoethanol, MEM nonessential amino acids, 1 mM sodium pyruvate, 2 mM l-glutamine (all Invitrogen, Paisley, UK), 10% (v/v) foetal bovine serum (SLS, Nottingham, UK) and 1 × 10^3^ U mL^−1^ leukemia inhibitory factor, LIF (Chemicon, UK). All stated concentrations are final. Cell dissociation was routinely achieved by removal of the culture medium followed by washing with Dulbecco's PBS (DPBS, Sigma, Poole, UK) and subsequent incubation for 4 min at 37 °C in the presence of 0.025% (w/v) trypsin supplemented with 0.372 g L^−1^ EDTA and 1% (v/v) chicken serum (all Sigma, Poole, UK). Following incubation the trypsinized cells were quenched using fresh culture medium and centrifuged for 3 min at 280 × *g* in an Eppendorf 5810R centrifuge in the case of manual cultures or in a Hettich Rotanta 46 RSC centrifuge in the case of automated culture. Following centrifugation and supernatant removal, the cells were resuspended in growth medium and replated onto a fresh gelatinized microtitre plate at the desired Inoculation Cell Density (ICD). Cultures were maintained in a humidified incubator at 37 °C with 5% (v/v) CO_2_.

### 2.2 Directed monolayer neuronal differentiation

ESCs were cultured manually for 2 days in serum containing medium in the presence of LIF as described in Section 2.1. Seeding at 3 × 10^4^ cells cm^−2^ in a T-25 flask typically yields around 6 × 10^6^ cells after 48 h. Differentiation was performed in Iwaki 6-well plates, the wells previously being gelatinized for around 1 h with 0.1% (w/v) gelatine at room temperature and then the excess gelatine removed. The cells were harvested by incubation with 0.025% (w/v) trypsin (containing 1% (v/v) chick serum) for ∼4 min as described in Section 2.1 and quenched with serum containing medium without LIF. The cells were centrifuged at 280 × *g* for 3 min, the supernatant removed and the pellet resuspended in LIF(-) medium. The cells were counted by Guava ExpressPlus™ (Guava Technologies, Lincolnshire, UK) flow cytometry and appropriately resuspended in LIF(-) medium containing serum to achieve a cell density of 5 × 10^3^ cells cm^−2^ and seeded in the plate overnight. The following morning all the serum containing medium was removed, the wells were washed with PBS (without calcium or magnesium), and NDIFF–RHBA medium (Stem Cell Sciences, Cambridge, UK) added. Medium was subsequently exchanged every 2 days, until the emergence of neuronal morphology (particularly axons) which was typically after 8–10 days. Cultures were maintained in a humidified incubator at 37 °C with 5% (v/v) CO_2_.

### 2.3 Embryoid body differentiation

ESCs propagated in T-flasks, following thawing of an Oct-4-GiP vial, were harvested as described in Section 2.1 and resuspended in serum containing medium in the absence of LIF. Approximately one tenth of the cells were reseeded into a 10 cm bacteriological petri dish (Sterilin, Caerphilly, UK) and further medium containing serum, without LIF was added. Medium was exchanged every 3–4 days. EBs were harvested after ∼8 days, washed with PBS (without calcium and magnesium) and stored at −80 °C for RNA extraction. Further EB differentiation experiments were performed on cells after undergoing 8 passages, either automated or manual, in 24-well plates.

### 2.4 Automation platform description

The automated pipetting station used in this study was a Tecan Freedom Evo 100 (Tecan, Reading, UK) equipped with one four-channel liquid handling arm and one gripper, RoMa, arm. This was housed in a Class 2 design biological safety cabinet (Walker Safety Cabinets, Derbyshire, UK), capable of environmental control (temperature, O_2_ and CO_2_ levels), and its operation integrated with a microplate centrifuge (Hettich Rotanta 46 RSC, Bach, Switzerland) and an automated CO_2_ incubator (Cytomat C450S, Basingstoke, UK) as shown in Fig. 1(a). The liquid handling arm was equipped with 1 mL syringes and was used with disposable tips only to minimize cross contamination. Control of the automated platform and the associated peripherals was through Evoware™ Standard (v2.4). A schematic of the automated platform deck indicating the location of on-deck devices, such as a microplate tilting rack and shaker, is shown in Fig. 1(b).

### 2.5 Automated Standard Operating Procedures (SOPs)

**Fig. 2:**
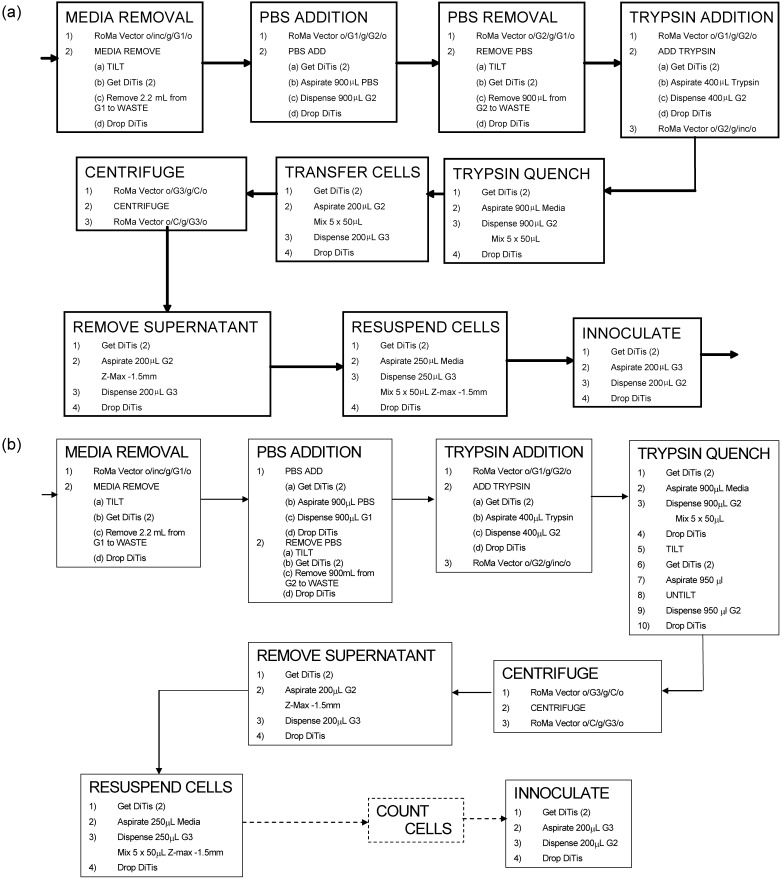
Detail of Standard Operating Procedures (SOPs) devised for the ‘hands free’ automated expansion and differentiation of mESCs; (a) single stage passage of adherent cell culture and (b) replating of cells for monolayer differentiation (dashed lines indicated manual cell count). Nomenclature: *o*: RoMa gripper open, *g*: Roma gripper closed, inc: CO_2_ incubator, *G*: Genesis tilting platform (positions numbered from back of the robot deck forwards), DiTi: disposable tip, and *Z*: vertical height of the pipetting arm.

The SOP devised for automated, hands-free ESC expansion is shown in Fig. 2(a). Before commencing each automated passage, all reagents were replenished and pre-warmed in the reagent trough rack by circulating warm water through the jacket. Source plate and destination plate locations were also inputted into the EvoWare™ software depending on the type of experiment being performed. Each culture plate was passaged after 2 days of growth which we have previously shown was sufficient to achieve >80% confluence and was optimal for maintaining the pluripotency of the ESCs [17]. Each plate was automatically removed from the incubator and was positioned by the RoMa arm in a predefined space on the plate rack and the lid removed. The rack was then tilted to 30° relative to the robotic deck and all media removed by aspiration. Pre-warmed DPBS was next added and the surface of the well washed by moving the rack between 0° and 30° and back five times. The DPBS was then removed, and warm trypsin added to each well. After ensuring the trypsin covered the surface, the plate was returned to the incubator for 5 min at 37 °C. Once the incubation was complete the trypsin was quenched using serum containing medium and the cell suspension mixed, by repeated aspiration and dispensing, to ensure efficient recovery of cells. The cell suspensions were then transferred to a 96-deep square well plate (ABgene, Epsom, UK) with conical bases for centrifugation. After centrifugation the supernatant was removed and the cell pellet resuspended in fresh medium. In the case of ‘fixed split’ cultures the cell suspensions were used to directly inoculate new cultures using a defined proportion of the cells; one sixth. For measured ICD cultures, the cell number was first determined manually using the Guava ExpressPlus™ assay described in Section 2.6.1, and the volume of cell suspension to be transferred entered into the EvoWare™ software.

Experiments to systematically optimize the automated passaging of cells via trypsinization examined a number of critical variables including trypsin incubation time, number of pipette mixing steps and aspiration/dispensing flow rates. Optimum trypsin incubation time was determined by exposing confluent 24-well microplate cultures to trypsin for various times after which quenching medium was added and the dissociated cell suspension removed. A proportion of the cell suspension was used to determine dissociated cell number using the Guava ExpressPlus™ assay described in Section 2.6.1. The remainder of the cell suspension was diluted by a factor of 5–10 depending on the cell number and replated in a 24-well plate. Aggregate size distribution was then determined by microscope inspection of 15 fields in 4 wells, per condition. The cells that remained attached were further treated with trypsin for 8 min to ensure all the cells were removed and the total cell number determined to allow for calculation of the dissociation efficiency. This is defined as the ratio of the number of viable cells detached in the first trypsinization to the total number of viable cells detached after the first and second trypsinization steps. Cell number and viability are presented as an average across one plate. Initial experiments showed that the well-to-well variation was minimal and was not affected by well location or sequence of passaging.

The SOP devised for automated monolayer ESC differentiation is shown in Fig. 2(b). Cells produced by microwell passaging were washed, dissociated and centrifuged as for a standard passage with the subsequent resuspension of the cells being carried out in serum containing medium without LIF. Cell number was then determined using the Guava ExpressPlus™ assay described in Section 2.6.1. A 6-well Iwaki microtitre plate pre-coated with 0.1% (w/v) gelatine was then inoculated with resuspended cells to an ICD of 5000 cells cm^−2^. Standard GMEM based medium containing serum but without LIF was added to give a final well volume of 2 mL medium. The plates were inoculated overnight and subsequently serum containing medium was removed and replaced with NDIFF–RHBA medium. Throughout the course of the differentiation, a complete medium exchange was automatically carried out every 48 h.

### 2.6 Analytical methods

#### 2.6.1 Cell density, viability and GFP expression

Viable cell concentration and cell viability were measured by staining 197 μL of cell suspension (diluted to achieve a cell concentration of 50–500 cells μL^−1^) with 3 μL of ViaCount™ stain (Guava Technologies, Hayward, CA, USA). After 10 min incubation the samples were analyzed on a Guava EasyCyte 96-well flow cytometer using Guava's ExpressPlus™ software. The viable population was gated based on co-staining with the ViaCount. GFP expression was determined using the PM3 channel of the Guava EasyCyte. A negative control for GFP expression was set up using the parental E14TG2a cell line [23].

#### 2.6.2 Quantitative RT-PCR

RNA was first extracted from cell pellets using the RNeasy Micro Kit (Qiagen, Sussex, UK) according to the manufacturer's instructions. The RNA was eluted in 12 μL of RNase-free water and the concentration was measured using a Nanodrop spectrophotometer (Thermo Scientific, Delaware, USA), measuring absorbance at wavelengths of 260 nm and 280 nm. Subsequently 1 μg of RNA was used to synthesize first strand cDNA using the RETROscript First Strand Synthesis Kit (Applied Biosystems, Cheshire, UK), again as per the manufacturer's instructions, using random decamers to prime the first strand reaction. To confirm successful reverse transcription, a standard RT-PCR reaction was carried out for the housekeeping gene *β-actin*. Reactions were carried out using the BIOTAQ PCR kit (Bioline Laboratories, London, UK) and the *β-actin* primers F-(seq) in a Veriti Thermal Cycler (Applied Biosystems, Cheshire, UK). PCR products were run in 2.5% agarose gel to confirm the presence of the desired 77 bp band corresponding to the correct β-actin amplicon.

For gene expression analysis 1 μL of the cDNA was used in a SYBR Green real time PCR reaction using the QuantiTect SYBR Green PCR Kit and the pre-validated primers QuantiTect Primer Assays (Qiagen, Sussex, UK) under the manufacturer's protocols, in a Mastercycler EP Realplex (Eppendorf, Stevenage, UK) thermocycler. Primer sequences are proprietary but details can be found at: https://www1.qiagen.com/GeneGlobe/Default.aspx. Primer assays were as follows: *Nanog* (QT01076334), *Oct3/4* (*Pou5F1*)(QT00109186), *Rex1* (*Zfp42*) (QT00299936), and *β-actin* (QT01136772). For relative quantification, the ΔΔ*C*_*t*_ method [24] was used. RNA levels were normalized against the housekeeping gene *β-actin* and cDNA from passage 1 cells was used as the calibrator. All calculations were performed using the realplex software (Eppendorf, Stevenage, UK).

#### 2.6.3 Immunocytochemistry

Differentiated mESCs maintained on gelatine coated tissue culture plastic were fixed using 4% (w/v) paraformaldehyde. After permeabilization and blocking, the cells were incubated for 1 h at room temperature with primary antibodies directed against brachyury, βIII-tubulin or alpha-fetoprotein (the primary antibody was raised in mouse). Secondary antibodies (anti-mouse) conjugated with Cy-3 were used to visualize the antibodies, and the cells were counter stained with DAPI. Negative controls were used where the primary antibody was omitted. The stained cultures were viewed using an inverted fluorescent microscope (Eclipse TE2000-U, Nikon, Surrey, UK). All antibodies were obtained from Abcam (Cambridge, UK).

## 3 Results and discussion

### 3.1 Framework for definition of Standard Operating Procedures

The next phase in the development of regenerative medicine bioprocessing will see the application of rigorous experimental design techniques [4] for the systematic analysis of factors influencing the quantity and quality of cells produced by a particular bioprocess [3,17,25]. The optimized factors and their levels will then need to be embedded within predefined SOPs that are amenable to automation such that they can be carried out reproducibly across multiple batches of cells. The SOPs must be devised such that, together with the chosen bioprocessing platform, they meet current regulatory guidelines [26] for ensuring the consistent production of cellular therapies that are safe for clinical application.

The initial selection of factors for investigation can be identified by a combination of statistical Design of Experiments (DoE) techniques [27] and an understanding of both stem cell biology [14,28] and biochemical engineering fundamentals that impact on the microenvironment to which cells are exposed [18,29–32]. For manufacturing purposes, and improved operational efficiency, these investigations are best embedded within a Quality by Design (QbD) approach [33]. In this work we began by systematically investigating over 10 factors that influence automated ESC growth and differentiation in microwell formats using a combination of DoE and traditional experimental approaches. The factors investigated included: culture ICD and duration, temperature, media pH, aspiration and dispensing flow rates, applied shear rate, trypsinization time, cell disaggregation conditions, relative centrifugal force, centrifugation time, and cell pellet resuspension rate. Initial factorial experiments showed that the use of default automation settings for trypsinization of cells led to a low yield of poor quality cells. This proved to be a key bioprocess step and so this is discussed further in Section 3.2. Centrifugation settings with centrifugal forces of 300 × *g*, 600 × *g*, and 1000 × *g* were evaluated in earlier work by Veraitch et al. [17]. Results showed that higher centrifugal forces had a negative effect on viable cell concentration, therefore a centrifugal force of 300 × *g* was used in this work. As noted by Kirouac and Zandstra [15] it will also be necessary to incorporate methods for assessing cell properties and their control within a particular SOP but these are beyond the scope of the current work.

### 3.2 Trypsinization optimization

The model cell line employed in this work is transfected with the *Oct-4-GFPirespac* transgene and expresses GFP under the control of the *Oct-4* promoter [34]. It has been shown that *Oct-4* is a key factor in the maintenance of pluripotency and cell fate [35–37]. GFP expression was therefore taken as an indicator of pluripotency during initial factorial experiments and prior to a more comprehensive analysis of gene expression. As mentioned above, the passaging of ESCs by trypsinization and centrifugation proved to be a key bioprocess step. The importance of trypsin removal was also noted by Liu et al. [38] who found that residual trypsin could lead to a reduction in the number of viable cells. Use of default values within the EvoWare™ software for parameters such as aspiration flow rate and position within a well resulted in low cell viabilities (<50%), poor cell yields (<80%) and the rapid loss of GFP expression (to <80%) after only two sequential passages. As a result of these observations, various aspects of the trypsinization process were further investigated for optimization purposes including: trypsin incubation time, aspiration flow rate, dispense flow rate and number of pipette mixing steps employed.

**Fig. 3:**
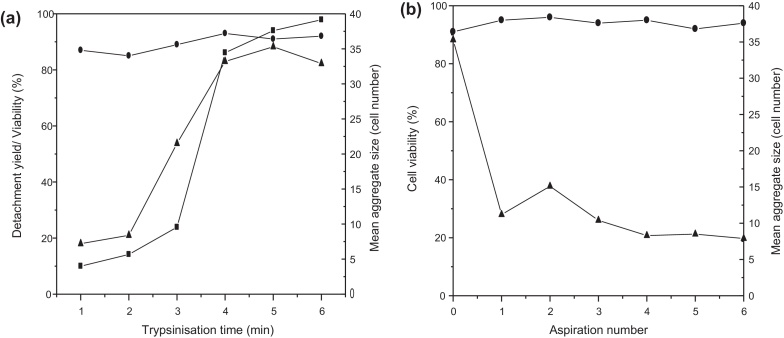
Optimization of cell detachment kinetics from a 24-well microplate. (a) The impact of trypsinization time on the detachment efficiency and viability of mESCs and the size of the aggregates removed. (b) The impact of the number of pipette aspirations (*u*_max_ = 0.3 ms^−1^) on the aggregate size and viability of the cells after 5 min exposure to trypsin at 37 °C: (■) cell detachment yield, (•) viability, and (▴) aggregate size. Experiments performed as described in Section 2.5.

The measured kinetics of cell detachment from the plate surface with respect to trypsin incubation time after a single passage is shown in Fig. 3(a). Trypsinization for a minimum of 4 min results in the detachment of over 95% of the cells with no significant effect on the measured cell viability. The *Oct-4*-GFP expression remained high, >90%, provided that the incubation time did not exceed 10 min, setting a critical upper limit on the process operating window. With regard to bioprocess reproducibility over multiple passages, it is noteworthy that the mean size of the detached cell aggregates also increased with incubation time (from approximately 5 to 40 cells per aggregate). Ideally, for reproducible inoculation of the next plate it would be desirable to obtain a single cell suspension at this stage. Visual observation indicated that at short incubation times only small ESC colonies were detached and while some discretely suspended cells were obtained, the cell yield was too low for the process to be viable. With increased incubation times the measured increase in cell yield and aggregate size was a result of a much greater portion of the cell monolayer becoming detached from the culture surface. For trypsin incubation times greater than 10 min, especially over multiple ESC passages, a more dramatic decrease in the final viable cell number (∼80%) and *Oct-4*-GFP expression (∼70%) was measured. In this case the use of automation helps ensure the consistent application of 4 min incubation times which can be difficult for a manual operator to achieve especially when passaging multiple plates in parallel.

Since the optimum trypsinization time for high cell yield and retention of *Oct-4*-GFP expression did not result in dissociation of the cell aggregates into a single cell suspension, the effect of ‘active pipetting’ of the aggregates to promote cell dissociation was investigated. Both the number of aspiration and dispense steps, as well as their respective flow rates were examined. The data obtained by increasing the number of aspiration steps following a 5 min trypsin incubation time is shown in Fig. 3(b). It was observed that a single aspiration and dispense step, at volumetric flow rates of 300 and 600 μL s^−1^, respectively, was sufficient to reduce the size of the released ESC aggregates to a mean value of just over ten cells per aggregate. However, the use of at least three aspiration and dispense steps resulted in the reproducible production of aggregates below ten cells per aggregate. As shown in Fig. 3(b) the use of these ‘active pipetting’ steps did not significantly affect the viability of the cells at the flow rates studied. Further investigation of the aspiration and dispense flow rates showed that an aspiration flow rate of 300 μL s^−1^ represented a threshold value, below which aggregate breakage was minimal, and above which no significant advantage was obtained (data not shown). Consequently in the SOP for the automated passaging of ESCs three aspiration (at 300 μL s^−1^) and dispensing (at 600 μL s^−1^) steps were included after addition of quenching medium.

### 3.3 Automated expansion of ESCs

Having identified the optimum factors and their settings for each operation in the automated passaging of the ESCs, these were incorporated within the SOP for cell expansion (Fig. 2(a)). The protocol involved all major steps routinely used in manual cell culture, including the centrifugation step which is important for trypsin removal [38]. In the manual culture process the plate was ‘tapped’ by hand to dislodge cells, a step which was replaced by ‘active pipetting’ in our SOP. Multiple, parallel microwell cultures were then performed over eight sequential passages.

The automated bioprocess was directly compared to a manual process. In each case both a fixed split strategy (wherein the cells are consistently split at a fixed ratio of the total cell number after 2 days culture) or by inoculation of subsequent passages at a measured ICD (requiring the cell number to be manually counted after each trypsinization and centrifugation step) was adopted. While it is possible to integrate automated cell counting within the platform such an approach is expensive and adds complexity. If reproducible ESC growth kinetics can be achieved between wells and from passage to passage then the fixed split strategy offers the benefits of a simpler bioprocess with reduced risk of equipment failure or contamination.

The first important metric to compare is the accuracy and precision in determination of the viable cell number at the end of a single passage. As shown in Table 1, the maximum coefficient of variance (CV) in the case of both automated bioprocess strategies is significantly less than for the equivalent manual process (based on four independent measurements of viable cell number from four separate wells). In the case of cells processed using the fixed split strategy, the maximum CV was 3.5% for a single automated passage, while for a single manual passage the maximum CV was 5.1%. In the case of cells processed by means of the measured ICD strategy the maximum CV values were lower, as expected given a more precisely defined inoculum, and the CV for the automated process was some 3-fold lower than for the manual one. These results confirm increased uniformity in plate inoculation and cell growth kinetics using an automated bioprocess. *Oct-4*-GFP expression levels over the single passage remain high with low CV values (Table 1) for both manual and automated processes giving an initial indication that the ESCs remain pluripotent.

**Fig. 4:**
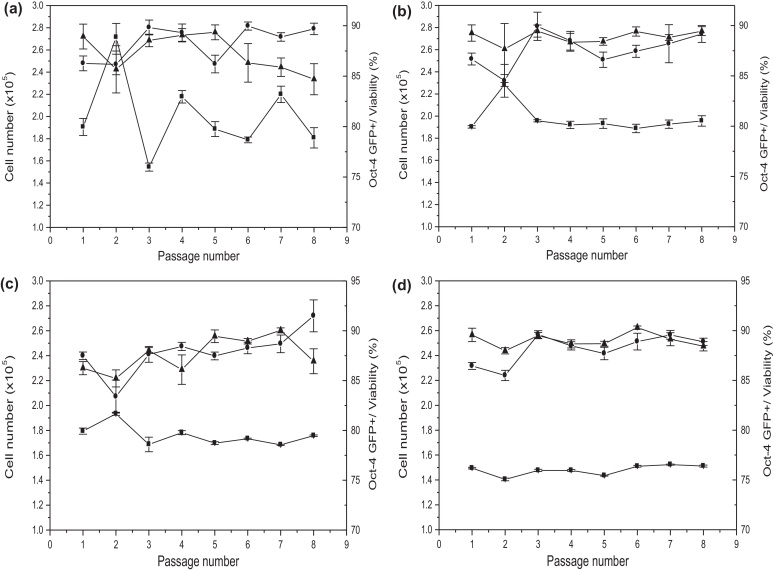
Comparison of manual and automated passaging of mESCs over multiple passages. Cells processed manually at (a) a fixed 1:6 (v/v) split ratio of the previous culture and (b) a measured ICD of 20,000 cells cm^−2^. Cells processed by automation platform at (c) a fixed 1:6 (v/v) split ratio of the previous culture and (d) a measured ICD of 20,000 cells cm^−2^: (■) cell number, (•) *Oct-4*-GFP+, and (▴) viability. Experiments performed as described in Section 2.5. Error bars represent 1 standard deviation about the mean (*n* = 4).

The ESCs were subsequently cultured over eight sequential passages again measuring the resulting cell number, *Oct-4*-GFP expression level and cell viability. Results for the processing of ESCs using a fixed split strategy for manual and automated bioprocesses are shown in Fig. 4(a) and (c), respectively. For the manual process significant variation in the final cell number is apparent from passage to passage. In contrast the measured cell number for the automated process remains considerably more stable. As shown in Table 1 over the eight passages, the calculated CV in cell number determination for the manual process is large at 17.8% while that for the automated process is some 3-fold lower at an acceptable level of 4.7%. Over the eight passages there was little difference in the overall viability of the cells with the manual process yielding an average cell viability of 88 ± 1.8% and the automated process yielding a value of 88 ± 2.2%. The mean *Oct-4*-GFP expression levels were similarly maintained in both manual (87 ± 1.9%) and automated processes (88 ± 1.8%) resulting in low and acceptable CV values (Table 1). The equivalent and high levels in cell viability and *Oct-4*-GFP expression after the eighth passage again suggest that the cells remain pluripotent and respond favourably to both manual and automated bioprocesses.

As shown in Fig. 4(b) and (d) the use of a measured and predefined ICD further improved the consistency and reproducibility of both manual and automated bioprocesses. For the manual process, apart from the one significant deviation in cell number at passage 2, the calculated CVs over the eight passages were low at 6.6% while that for the automated process showed a further 2-fold decrease (Table 1). Again, there was little difference in the measured cell viabilities (∼88%) and *Oct-4*-GFP expression levels (∼89%) in either case. Irrespective of the operating strategy employed (fixed split or manual ICD) these results confirm that compared to manual cultures performed by a well trained and experienced researcher the application of automation results in the more reliable and predictable production of cells of consistent viability and pluripotency.

**Fig. 5:**
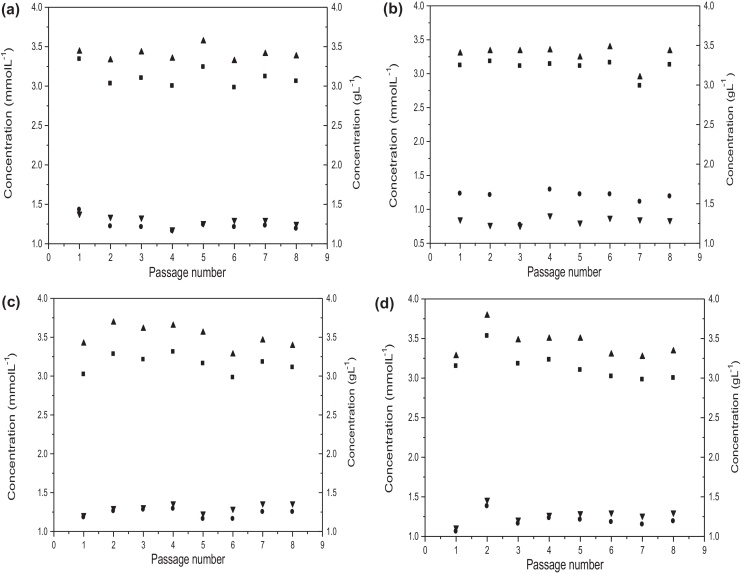
Extracellular metabolite analysis after 2 days culture post passage of cells produced during the manual and automated cultures shown in Fig. 4: (■) l-glutamine (mmol L^−1^), (▴) glucose (g L^−1^), (▾) lactate (g L^−1^), (•) ammonium (mmol L^−1^). Cells passaged using the methods described in Fig. 4: (a) manual, fixed split (b) manual, measured ICD (c) automated, fixed split and (d) automated, measured ICD. Metabolites measured as described in Section 3.3.

The spent medium for each passage after 2 days culture was analyzed for key metabolite levels as shown in Fig. 5. In the majority of cases the absolute levels of the metabolites were similar suggesting that the application of automation has no measurable effect on the metabolic activities of the ESCs under investigation. The metabolite levels are also consistent from passage to passage which is expected given the similar levels of viability and cell growth observed (Fig. 4). In future, analysis of metabolic consumption and production rates would allow us to further evaluate any metabolic differences between automated and manual processing.

Bioprocess reproducibility will ultimately underpin all aspects of successful and cost effective manufacture of cells either for drug discovery or therapy. In this regard, it should also be noted that automated ESC culture over extended passages has now been performed on multiple occasions in our laboratory over a 6 month period. Even in the absence of selective antibiotics in the culture media there has not been one incidence of culture contamination during contained operation of the robotic platform. There are a number of advantages to processes carried out in the absence of antibiotics, which the automation platform facilitates. Primarily, the growth rate and differentiation capacity of ESCs has been shown to be reduced in the presence of antibiotic agents [39], thereby significantly increasing culture times and adversely affecting space-time yields. In addition, the widespread use of antibiotics in stem cell culture has been shown to mask contamination [40], presenting a potential risk in the use of cell therapies derived through cultures employing antibiotics.

### 3.4 Gene expression analysis during ESC expansion

Quantitative real-time PCR was carried out to establish any differences in the expression levels of pluripotency markers between cells passaged manually and those passaged using the robot. Representative expression levels (from triplicate experiments) at passages 1, 5 and 8 were used to determine the effect of the culture method on the pluripotency of the cells. The relative expression levels of the genes *Oct-4*, *Nanog* and *Rex-1* were determined for each of the bioprocess strategies described in Fig. 4. The sensitivity of *Rex-1* to small changes in the pluripotent state of ESCs has been previously described [41] and so it was hypothesized that any significant changes in the pluripotency of the cells caused by automated processing would result in changes in the level of Rex-1 expression. Use of *Oct-4* and *Nanog* are standard in the ESC literature [29,42,43] and were used to further confirm the pluripotency of the cells after culture.

**Fig. 6:**
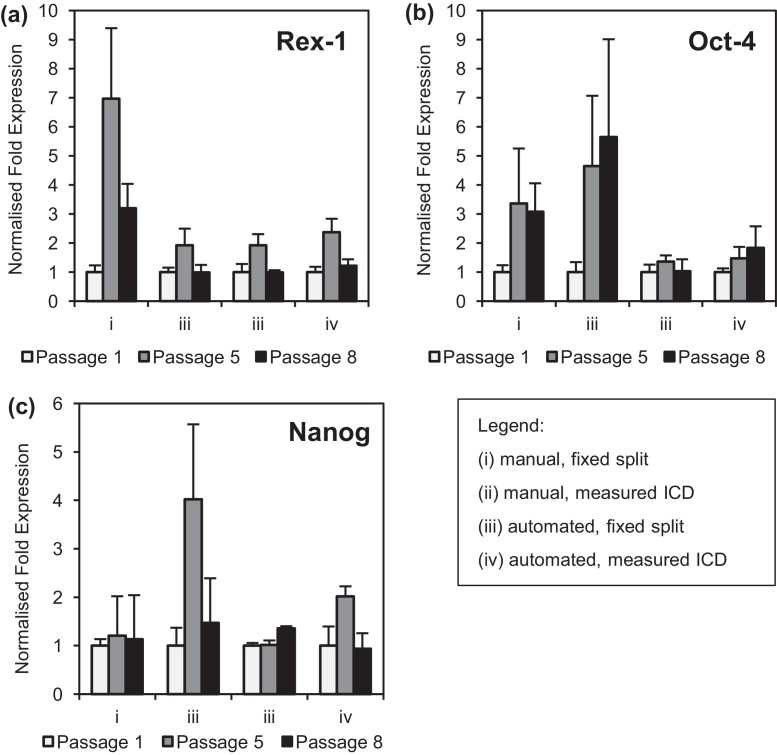
Quantitative real-time PCR analysis of the cells produced during the manual and automated cultures shown in Fig. 4. Graphs show the relative expression of (a) *Rex-1*, (b) *Oct-4*, and (c) *Nanog*, at passages 1, 5 and 8 using various bioprocess strategies: (i) manual, fixed split; (ii) manual, measured ICD; (iii) automated, fixed split and (iv) automated, measured ICD. Error bars represent 1 standard deviation about the mean (*n* = 3). PCR performed as described in Section 2.6.2.

Analysis of *Rex-1, Oct-4* and *Nanog* expression, normalized against *β-actin* expression as a housekeeping gene, is shown in Fig. 6(a)–(c), respectively. There is some variation in the data. For plutipotency markers *Rex-1* and *Nanog* the level of gene expression is reasonably similar between manual and automated methods as well as with passage number. In the case of cells processed manually with a fixed split ratio, *Rex-1* expression was variable with that at the end of passage 5 seven times that at passage 1. For cells processed on the automation platform with a similar split regime, the *Rex-1* levels after 8 passages are very similar at two times that observed at passage 1. A similar pattern of *Rex-1* expression was observed in both cases of cells inoculated at a measured ICD. However, there is large variation in *Oct-4* expression during manual processing as indicated by the error bars. In general, results from automated cultures showed pluripotency marker expression was much more consistent compared to manual passages. The maintenance *Rex-1, Oct-4* and *Nanog* in mESCs indicates the amenability of the cells to automated bioprocessing and the application of consistent and reproducible process operating conditions between wells and between passages. To further evaluate the quality of cells after automated processing, assays such as western blot analysis and PCNA staining would be necessary to determine protein levels and assess whether there are differences in cell proliferation.

### 3.5 Embryoid body differentiation of ESCs produced by automation

**Fig. 7:**
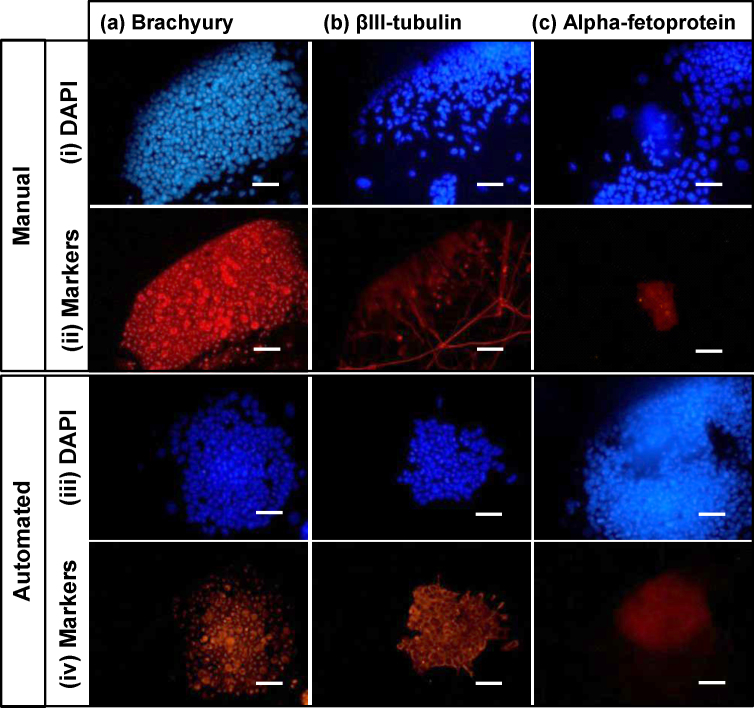
ICC analysis of embryoid bodies created after 8 passages through (i and ii) manual and (iii and iv) automated bioprocess strategies for (a) brachyury, (b) βIII-tubulin and (c) alpha-fetoprotein. Blue images (i and iii) show DAPI staining and red images (ii and iv) show actual markers. Scale bars 100 μm. ICC performed as described in Section 2.6.3.

In order to confirm that the cells produced on the automation platform remained pluripotent, the cells harvested after passage 8 were subjected to a manual EB differentiation protocol. Results shown in Fig. 7 indicate that cells processed on the automated platform maintained their ability to differentiate into cells of the mesoderm, ectoderm and endoderm lineages. Cells produced by both manual and automated processing stained positive for brachyury (mesoderm), βIII-tubulin (ectoderm) and alpha-fetoprotein (endoderm). These results confirm the maintenance of pluripotency during cell expansion as shown in Section 3.4.

### 3.6 Automated directed differentiation of ESCs

The preceding results have illustrated the successful ‘hands-free’ expansion of mESCs over multiple passages and the maintenance of high levels of cell viability and pluripotency gene expression. Although expansion of ESCs in an automated fashion is desirable and required, the most likely ‘end-user’ requirements for ESCs will be defined cell populations in various stages of differentiation. This requires not only a robust and reproducible method for producing pluripotent ESCs in bulk (as described in Section 3.3) but also an equally robust method for the subsequent directed differentiation of the resultant cells to desired lineages, or specific cell types. One of the cell types offering most promise in the area of drug discovery and regenerative medicine are neuronal cells [4] and so were the focus of the differentiation experiments here.

An SOP for automated monolayer neuronal differentiation was established in a similar manner to that for cell passaging (Fig. 2(a)). Many of the optimized factor settings used in the cell expansion SOP, such as definition of the plate tilt angle and aspiration location for maximum removal of spent culture medium from a well, could be directly translated to the SOP for monolayer differentiation (Fig. 2(b)). Further optimization was needed in certain areas however. For example, it was necessary to separately determine maximum aspiration (150 μL s^−1^) and dispensing (300 μL s^−1^) flow rates for cell suspensions containing cells with significant neurite outgrowth. This being a consequence of their greater sensitivity to the hydrodynamic and shear forces experienced during pipetting [44].

**Fig. 8:**
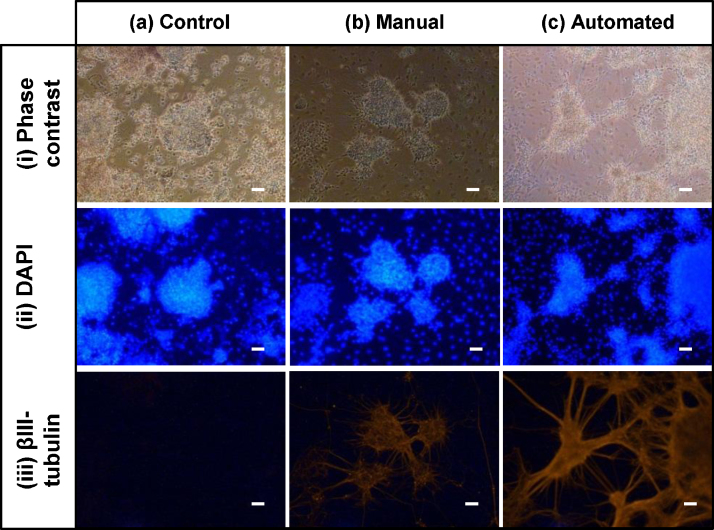
ICC analysis of representative mESCs after 10 days of monolayer differentiation. (a) control; secondary antibody staining only (b) manual differentiation, and (c) automated differentiation. (i) Phase contrast, (ii) DAPI and (iii) βIII-tubulin staining. Scale bars 100 μm. ICC performed as described in Section 2.6.3.

**Fig. 9:**
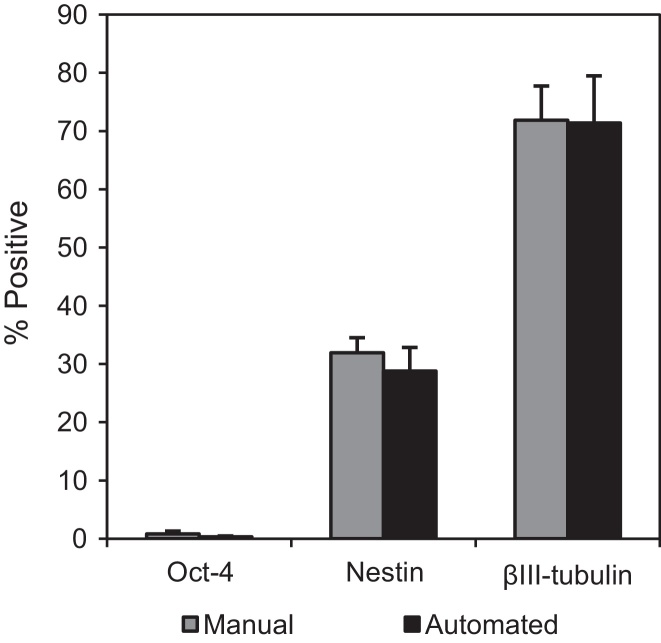
Flow cytometry of mESCs at day 10 of manual and automated differentiation for pluripotency marker *Oct-4* and neural markers *Nestin* and *βIII-tubulin*. Expression of *Oct-4* on day 0 for automated and manual processing was 90% while *Nestin* and *βIII-tubulin* were negligible. Error bars represent 1 standard deviation about the mean (*n* = 3).

Once established, the SOP for the monolayer differentiation protocol enabled the optimized factor settings and ranges to be reproducibly applied across parallel microwell monolayer differentiation experiments. Again the automated differentiation protocol using NDIFF–RHBA media was compared to the standard manual differentiation process described in Section 2.2. The differentiated cells produced were analyzed by immunocytochemistry, and the results are displayed in Fig. 8. It can be seen from the phase contrast images that the cell morphology is as expected with neuronal rosettes present with divergent axons. The presence of the late neuronal marker βIII-tubulin [34,45,46] is confirmed in both the manually processed cells and the cells processed using the automated SOP. The absence of any staining in the case of the control (Fig. 8(a, iii)), indicates that there was no non-specific binding of the secondary antibody confirming that the observed activity is due to the presence of βIII-tubulin. Cells were also stained and analyzed using flow cytometry (Fig. 9) with the results confirming those seen in Fig. 8. Levels of neural markers Nestin and βIII-tubulin expressed during manual and automated differentiation were found to be comparable. As neuronal cells can be sensitive to processing, automated bioprocessing may be advantageous as it allows shear forces to be controlled more consistently than in manual bioprocessing. Being able to control the pipetting speed accurately, for example, could reduce damage to the cells during processing. In future work it would therefore be beneficial to use a functional assay on the neuronal cells differentiated on the platform and compare the results to cells differentiated manually.

## 4 Conclusions

This study has described an integrated automation platform designed for the ‘hands-free’ culture of ESCs over multiple passages and their subsequent directed differentiation into neuronal precursors. Automated processing of the ESCs showed enhanced reproducibility and predictability compared to manual methods, while maintaining the cells’ pluripotency and ability to differentiate into the three embryonic germ layers. It also illustrated a successful framework for the establishment and optimization of SOPs for automation platform operation. The results clearly demonstrate that the automated bioprocess outperforms the manual one even when the latter is performed by a highly trained and experienced researcher. The improved reproducibility of the automated process enables simpler bioprocess strategies to be adopted, such as the use of fixed split ratios between passages, increasing throughput and reducing the risk of equipment failure or contamination. This study thus confirms the benefits of adopting automated bioprocess routes to produce cells both for therapy and for use in basic discovery research. Current work in our laboratory is investigating the control of culture conditions, such as oxygen levels, to improve the yield and purity of differentiated cell types and the expansion of human ESCs on the robotic platform.

## Figures and Tables

**Table 1:** Statistical reproducibility of manual versus automated culture of mESCs. Data taken from Fig. 4.

Process metric	Maximum coefficient of variance (%)			
	Measured ICD		Fixed split	
	Manual	Automated	Manual	Automated
Viable cell count (1 passage)	2.4	0.8	5.1	3.5
Viable cell count (8 passages)	6.6	2.7	17.8	4.7
Oct-4-GFP expression (1 passage)	2.9	0.8	2.7	1.7
Oct-4-GFP expression (8 passages)	1.3	0.9	2.1	2.0
